# Patterns of thoracic injury in bomb blast victims: A retrospective radiological review

**DOI:** 10.1177/03000605251385839

**Published:** 2025-10-21

**Authors:** Muhammad Nadeem Ahmad, Muhammad Abdullah, Reyan Hussain Shaikh, Ramsha Pervez, Mallick Muhammad Zohaib Uddin, Naila Nadeem, Arsalan Saleem, Uffan Zafar

**Affiliations:** 1 Department of Radiology, Aga Khan University Hospital, Pakistan; 2 Medical College, Aga Khan University Hospital, Pakistan; 3 Diagnostic and Interventional Radiology, 12338 The University of Texas Medical Branch at Galveston, USA

**Keywords:** Bomb blast, thoracic injury, blast trauma, mass casualty incident, retrospective study, emergency radiology, thoracic radiology

## Abstract

**Introduction:**

Bombings, accounting for approximately 50% of global terrorist incidents, frequently cause high-morbidity thoracic trauma, including blast lung injury. This retrospective radiological review characterizes injury patterns in bomb blast victims to guide mass casualty response and improve patient outcomes.

**Methods:**

This retrospective observational review, conducted at Aga Khan University Hospital (January 2004–October 2024), included 130 patients with bomb blast injuries. Demographics, injury mechanisms, and imaging findings were categorized by blast type and summarized using frequencies, percentages, medians, and interquartile ranges.

**Results:**

Among 130 victims (94.6% males; median (interquartile range) age, 32 (26.0–43.5) years), initial chest X-ray was performed in 85.4% of cases, detecting foreign bodies (22.8%), emphysema (10.4%), and atelectasis (10.4%). Computed tomography was performed in 28.5% of the patients on the second imaging assessment; however, foreign bodies and atelectasis persisted at 14.4%–15.9% on follow-up. Primary blast injuries predominated (68.4%–78.8%), followed by secondary (15.0%–23.3%), tertiary (0%–4.7%), and quaternary (1.8%–4.4%) injuries; additionally, 48.5% of patients did not undergo a third study.

**Conclusions:**

Primary blast injuries predominate, with frequent foreign bodies, emphysema, and atelectasis. Initial chest X-ray facilitates rapid assessment, while computed tomography is reserved for complex cases. Tailored imaging protocols may enhance timely care and outcomes in resource-limited settings.

## Introduction

Terrorism is broadly defined as a method of coercion that employs violence to spread fear and achieve political or ideological goals.^1^ In 2019, 8473 terrorist incidents were reported worldwide, resulting in 20,309 deaths.^2^ Longitudinal data spanning nearly two decades (1983–2002) from the United States documented 36,110 bombing incidents, resulting in 5931 injuries and 699 fatalities.^3^ In contrast, low- and middle-income countries, such as Pakistan, bear a heavier burden, with approximately 10,000 deaths reported between 2007 and 2016.^4^ Despite a global decline in terrorism-related deaths, certain regions such as South Asia continue to face persistent threats, reflecting localized surges in militant activity. According to the annual Global Terrorism Index, the number of countries affected by terrorist attacks increased from 58 to 66, reversing nearly a decade of progress.^5^ Although terrorism manifests in different forms, bombings constitute a significant proportion, accounting for 48.57% of incidents worldwide between 1970 and 2017.^6^

Beyond its staggering death toll, terrorism inflicts lasting physical and psychological distress on survivors. Bombings, in particular, cause severe injuries that often require extensive medical care. Among the primary injuries sustained in such attacks, blast lung injury (BLI) has emerged as a predominant concern, affecting approximately 24% of victims, with morbidity rates reaching as high as 88.3%.^7^ BLI primarily affects gas-filled organs, including the lungs, bowel, and middle ear, making the thoracic region particularly vulnerable to severe trauma.^8^ Affected individuals are at increased risk of developing life-threatening complications in the thorax region, such as pneumothorax, hemothorax, lung contusions, and atelectasis.^9^ The ensuing respiratory distress often requires mechanical ventilation and intensive care management. In severe cases, BLI can progress to acute respiratory distress syndrome, leading to respiratory failure. Despite intensive treatment, the prognosis remains guarded, with survivors often experiencing prolonged hospitalization and long-term pulmonary dysfunction.^8^

Given the high prevalence and severity of thorax injuries in bomb blast incidents, understanding their patterns is essential for effective clinical management. By precisely characterizing thoracic injury patterns, we can develop targeted emergency protocols, perform rapid diagnosis through effective imaging modalities, and implement necessary interventions urgently. This radiological review outlines key injury findings associated with bomb blast–related thoracic trauma to tailor the emergency response strategies and guide timely clinical interventions, thus improving patient outcomes in these high-stakes scenarios.

## Material and methods

### Study design and setting

This retrospective observational radiological study was conducted at Aga Khan University Hospital (AKUH), Karachi, Pakistan, from 1 January 2004 to 31 December 2024. The study included all patients admitted consecutively with bomb blast injuries who underwent at least one radiological assessment of the thorax, including plain chest radiograph, chest computed tomography (CT), or thoracic ultrasound, performed as part of their clinical care. Patients with incomplete medical records, primarily imaging findings, or those without thoracic imaging were excluded. This study has been reported in accordance with the Strengthening the Reporting of Observational Studies in Epidemiology (STROBE) guidelines.^10^

### Radiological examinations

Upon arrival at the emergency department, all patients underwent immediate triage as part of the institutional mass casualty incident protocol. Initial radiological evaluation was performed at the discretion of the attending emergency physician and trauma team, guided by the patient’s hemodynamic stability and clinical findings.

The most common primary imaging modality for initial assessment was plain chest radiography. Standard anteroposterior (AP) chest radiographs were used to rapidly identify life-threatening thoracic injuries. Follow-up imaging (typically performed within 24–72 h after admission) was performed based on clinical indications such as worsening respiratory status or equivocal initial findings.

Contrast-enhanced CT of the chest was performed for patients with complex injuries, suspected vascular trauma, or inconclusive radiographic findings. For this retrospective analysis, we recorded and analyzed the findings from up to three sequential imaging studies for each patient to characterize the evolution of thoracic injuries. Subsequent imaging beyond index studies was performed only when clinically warranted by the patient’s ongoing medical course. The timing of these examinations was not determined by a standardized protocol or performed at fixed intervals; instead, it was dictated by the clinical progression and therapeutic requirements of each individual patient.

### Operational definitions

Following radiological review, all blast-related injuries for each patient were classified according to established criteria for blast injury mechanisms.^11^ The injuries were categorized into the following four types:

1. Primary injuries: These refer to injuries resulting from the direct barotrauma induced by the high-pressure blast wave. These predominantly affect gas-containing organs, such as the lungs (blast lung) and gastrointestinal tract.
2. Secondary injuries: These refer to injuries resulting from penetrating or blunt trauma caused by projectiles accelerated by the explosion. These projectiles include fragments from the explosive device (e.g. shrapnel) as well as environmental debris.
3. Tertiary injuries: These refer to injuries caused by the violent displacement of the victim’s body by the blast wind, leading to high-energy impact with the ground or stationary objects, or injuries sustained from structural collapse.
4. Quaternary injuries: These refer to all other injuries, illnesses, or complications that are not attributable to the first three mechanisms. This category includes thermal and chemical burns, inhalation of toxic substances, or exacerbations of pre-existing medical conditions such as angina or chronic obstructive pulmonary disease.

### Data collection and statistical analysis

As this was a retrospective review, no sample size calculation was required. Demographic and radiological data were retrieved from the hospital’s information management system after obtaining approval from the ethical review board and were analyzed. Radiological assessments were categorized into three sequential examinations based on the order in which they were performed during hospitalization, labeled as first radiological examination, second radiological examination, and third radiological examination. These findings were recorded based on official reports from the hospital’s radiology department.

Statistical analyses were primarily descriptive, performed using R statistical software, version 4.4.2 (R Foundation for Statistical Computing, Vienna, Austria). Categorical variables were presented as frequencies and percentages (%) and continuous variables as medians with interquartile ranges (IQRs). For injury pattern analysis, each distinct injury identified on radiological review was treated as an individual event. This approach acknowledges that a single patient could sustain multiple injuries across different categories (e.g. primary and secondary). The frequency of each specific radiological finding (e.g. pneumothorax and foreign body) was calculated as a proportion of the total patient cohort. The distribution of injury mechanisms (primary, secondary, tertiary, and quaternary) was calculated as a percentage of the total number of classified injuries.

### Ethical considerations

The study protocol was granted an exemption from formal review by the Ethical Review Committee (ERC) of Aga Khan University (Reference: 2024-9956-32023), considering the retrospective design and the use of fully anonymized patient data. The study was conducted in accordance with the ethical principles outlined in the Declaration of Helsinki, as revised in 2024. Patient confidentiality was maintained at all stages, as all data were deidentified prior to analysis.

## Results

### Patient demographics and hospital presentation

The cohort included 130 patients, primarily consisting of males (123; 94.6%), with only 7 females (5.38%). The patient ages ranged from 8 to 80 years (median (IQR), 32 (26–43.5) years). A majority (n = 71; 54.6%) of the patients presented directly to the emergency department of Aga Khan Hospital, 40.8% (n = 53) sought prior care elsewhere, and 4.62% (n = 6) had an unknown status. All patients underwent at least one radiological evaluation, with up to three sequential imaging stages analyzed.

Chest radiography was the primary initial imaging modality (85.4%, n = 111), followed by CT (n = 18; 13.8%). Subsequent evaluations revealed increased CT utilization (n = 37; 28.5%) in secondary imaging, although chest radiographs remained prevalent (n = 48; 37.7%). By the third assessment, chest radiographs remained the dominant modality (56; 43.1%), while approximately half of the participants (n = 63; 48.5%) required no further imaging.

Details of radiological examinations in the context of thoracic injuries are provided in Table 1.

**Table 1.** Frequency of imaging modalities used in the examination of bomb blast victims.

	Count (n)	Percentage (%)
First radiological examination		
Chest radiograph	111	85.4
Chest CT	18	13.8
Ultrasound	1	0.77
Second radiological examination		
Chest radiograph	49	37.7
Chest CT	37	28.5
Ultrasound	2	1.54
CT angiography	1	0.77
NA	41	31.5
Third radiological examination		
Chest radiograph	56	43.1
Chest CT	10	7.69
Ultrasound	1	0.77
NA	63	48.5

CT: computed tomography; NA: not available.

### Radiological findings

Initial radiological assessment revealed diverse thoracic abnormalities. Notably, foreign bodies were most frequently observed (n = 44; 22.8%), followed by emphysema (n = 20; 10.4%) and atelectasis/collapse (n = 20; 10.4%). Pneumothorax and lung contusions each occurred in 7.25% (n = 14) of cases, while pleural effusion was identified in 8.81% (n = 17). Other less frequently observed findings (<10%) included hemothorax, pulmonary haziness, arterial insufficiency, diaphragmatic injury, and various fracture patterns. It is important to note that 46 bomb blast victims (35.4%) had no significant thoracic findings in their initial radiologic assessment.

The second imaging assessment was largely consistent with the initial assessment, with persistent foreign bodies (n = 26; 14.4%) and atelectasis/collapse (n = 26; 14.4%) as the most frequent findings, followed by emphysema (n = 15; 8.33%). Complications included pneumothorax (n = 18; 10%) and subcutaneous emphysema (n = 6; 3.33%). Additionally, 61 patients (46.9%) had no thoracic findings on the second imaging assessment. However, 41 patients (31.5%) did not undergo a follow-up radiological examination.

The findings of the third imaging assessment mirrored those of the previous two examinations, but in fewer patients. Foreign bodies and atelectasis/collapse remained the most common thoracic findings (n = 18; 15.9% each). Pleural effusion and lung contusions persisted in 16 (14.2%) and 12 (10.6%) patients, respectively. Other findings included emphysema (n = 9; 7.96%) and pneumothorax (n = 10; 8.85%), each accounting for <10%. Eighty-one patients (62.3%) had no thoracic findings on the third imaging assessment, while approximately half of the patient population (48.5%, n = 63) did not undergo a third radiological examination.

**Figure 1.**
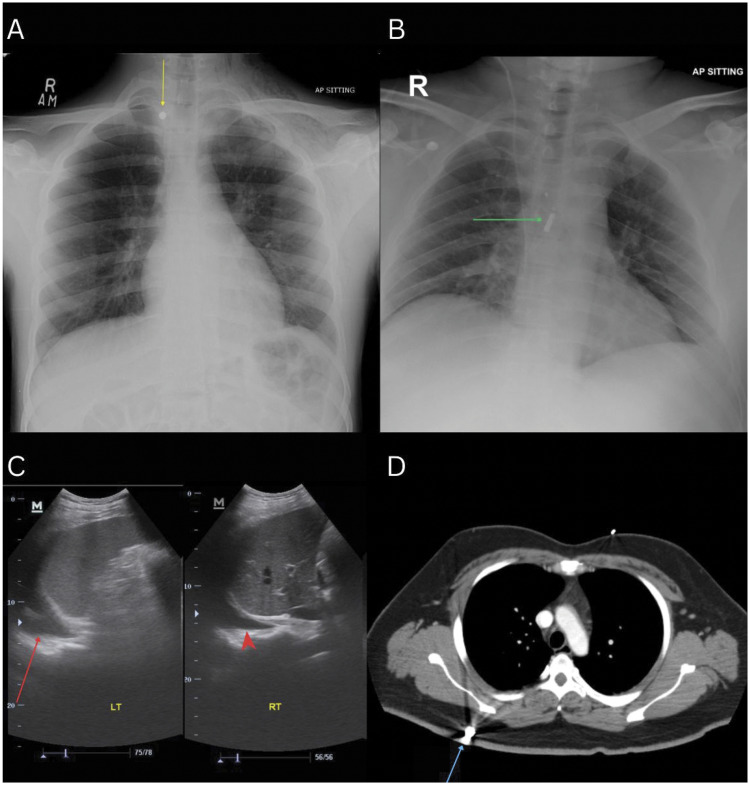
Multimodality imaging of thoracic and associated injuries in bomb blast victims. (a) AP CXR shows a hyperdense metallic foreign body (yellow arrow) overlying the medial aspect of the right clavicle. Subcutaneous emphysema is visible along the left thoracic wall, and a band-like opacity in the left upper lobe is consistent with a pulmonary contusion. (b) AP CXR demonstrating a large metallic foreign body (green arrow) projected over the thoracic spine. (c) Focused Assessment with Sonography in Trauma (FAST) of the left upper quadrant revealing left-sided pleural effusion (red arrow), with the second imaging assessment demonstrating a right-sided pleural effusion (red arrowhead) (d) axial contrast-enhanced CT image of the chest demonstrating distinct metallic foreign bodies (blue arrows) lodged within the subcutaneous soft tissues of the right posterior chest wall. CT: computed tomography; AP: anteroposterior; CXR: chest X-ray.

Figure 1 illustrates the spectrum of thoracic injuries and the multimodality imaging approach utilized in our patient cohort. Representative examples include secondary blast injuries, such as penetrating metallic foreign bodies identified on radiography and CT, as well as evidence of primary BLI and associated abdominal trauma.

**Figure 2.**
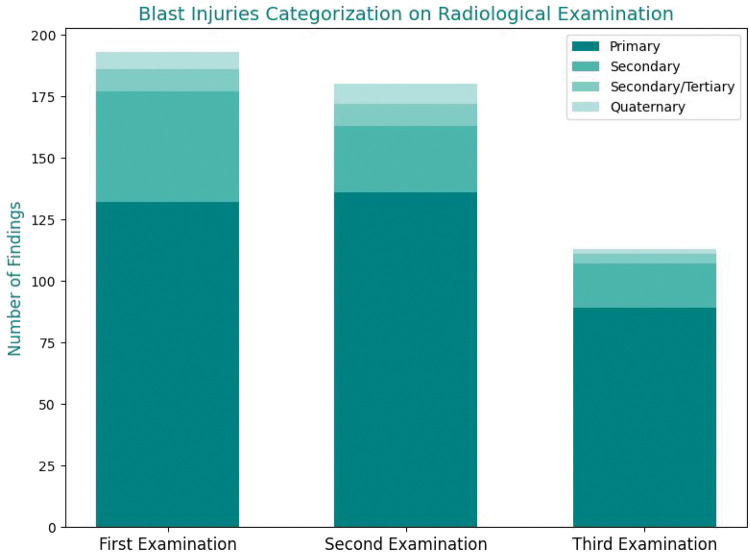
Distribution of blast injury categories across three sequential radiological examinations.

Figure 2 illustrates the distribution of blast injury categories across three sequential radiological examinations. All radiological findings were classified as primary, secondary, secondary/tertiary, or quaternary injuries. In the initial examination, primary blast injuries accounted for 68.4% (n = 132) of the documented findings, followed by secondary injuries (23.3%; n = 45), secondary/tertiary injuries (4.7%; n = 9), quaternary injuries (3.6%; n = 7), and no tertiary injuries. In the second examination, the frequency of primary injuries increased to 75.6% (n = 136); the frequency of secondary injuries was 15.0% (n = 27), that of secondary/tertiary injuries was 5.0% (n = 9), and that of quaternary injuries was 4.4% (n = 8), with no tertiary injuries identified. Similarly, the third examination revealed that primary injuries remained predominant at 78.8% (n = 89), followed by secondary injuries at 15.9% (n = 18), secondary/tertiary injuries at 3.5% (n = 4), and quaternary injuries at 1.8% (n = 2), with no tertiary injuries reported. These data consistently demonstrate the predominance of primary blast injuries across all examinations, with minor fluctuations in the other categories.

## Discussion

Blast injuries can cause life-threatening complications, often presenting with diverse injury patterns across multiple organ systems.^12^ Several factors influence these patterns, including the blast environment (open vs. closed space), the composition and quantity of the explosive material, and the victim’s proximity to the blast.^13,14^

The predominance of males (94.6%) in our cohort, consistent with previous studies from Pakistan, likely reflects the country’s sociocultural and occupational factors.^15^ Males are more frequently present in public spaces, workplaces, and high-risk areas where blasts typically occur.^16^ The wide age range, with a median age of 32 years, suggests that working-age adults are disproportionately affected due to increased outdoor exposure. A significant proportion of patients (54.6%) presented directly to AKUH, likely reflecting its reputation as a tertiary care center with advanced trauma facilities. Furthermore, 40.8% of the patients initially sought care elsewhere, possibly due to the proximity to other healthcare centers, initial stabilization at smaller hospitals, or transport delays.

Blast injury patterns are classified into four categories: primary, secondary, tertiary, and quaternary injuries.^11^ Primary injuries result from the direct impact of blast waves, primarily affecting gas-filled organs such as the lungs and gastrointestinal tract. Secondary injuries arise from shrapnel, soil, and other debris propelled by the blast wave. Tertiary injuries result from the displacement of victims by the blast wind or structural collapse, while quaternary injuries include burns and inhalation of toxic substances.^11^

In our study, primary blast injuries emerged as the predominant mode of injury and the most frequent radiological finding among blast victims across all three assessment modalities. This contradicts the existing literature, which emphasizes secondary blast injuries as more common and the leading cause of mortality in explosion victims.^17^ This discrepancy may be attributed to the unique characteristics of the blast events in our setting, environmental factors (e.g. confined spaces or proximity to the epicenter), or the type and composition of explosives used, which may have amplified overpressure effects, increasing the incidence of primary injuries. Furthermore, the lower detection of secondary injuries may reflect the limitation of restricting imaging to thoracic radiological assessments, potentially underestimating injuries in other anatomical regions, such as the extremities or head and neck.

The varied clinical presentations of blast injuries necessitate prompt and accurate diagnosis to guide timely management. Imaging plays a crucial role in injury detection; chest X-ray (CXR) is considered the first-line imaging modality worldwide owing to its cost-effectiveness, lower radiation exposure compared with CT, and rapid image acquisition.^18,19^ Our study corroborates this finding, with CXR serving as the initial imaging modality in 85.4% of cases. In low-resource settings such as Pakistan, CXR remains widely accessible, further explaining its predominant use. Despite the widespread use of CXR, CT is significantly more sensitive in detecting chest injuries^20–22^ and demonstrates superior efficacy in identifying pathologies of the chest wall, diaphragm, airways, heart, and esophagus.^23^ However, CT is limited by the higher costs and increased radiation exposure, despite its effectiveness as a diagnostic tool.^24^

Lung contusions represent one of the most common parenchymal injuries resulting from blunt injury to the chest.^25^ Studies have shown that CT is more sensitive than CXR in detecting pulmonary contusions.^26^ Traub et al.^27^ reported detection rates of 16% for CXR and 31% for CT, while Wakeel et al.^20^ revealed rates of 31% and 45%, respectively. Unlike CXR, which may require 6 h for contusions to become visible, CT detects them immediately after injury.^28^ However, early CT-detected contusions that are not visible on CXR have limited clinical significance and do not alter treatment.^25,29^ In our study, lung contusions were observed in 10.8% of cases on initial imaging and 9.2% on the third imaging assessment. Despite increased CT use on the second imaging assessment, detection rates remained low, suggesting that CXR can overestimate contusions due to lower specificity, while CT offers more precise evaluation, potentially ruling out false positives from initial CXRs. Importantly, contusions involving one-third of the pulmonary air spaces may necessitate mechanical ventilation, although this was not a predominant finding in our cohort.^30^

Pneumothorax is another frequent complication following blunt and penetrating traumatic injury. In blast victims, AP CXR is typically performed but may miss up to 50% of pneumothoraces.^31^ This is a significant limitation, as even small pneumothoraces can be clinically relevant, particularly in patients requiring positive-pressure ventilation, where there is a heightened risk of tension pneumothorax.^32^ In our study, pneumothorax was initially detected in 10.8% of cases, with a slight increase in frequency on the second imaging assessment (13.8%) when more patients underwent CT. However, during the third imaging assessment, when CXR was again the predominant modality, the detection rate dropped below 10%. This fluctuation suggests that pneumothoraces may have been underestimated during the initial and third assessments due to reliance on AP CXR. The higher detection rate on the second imaging assessment reinforces CT’s superior sensitivity, highlighting its importance in trauma settings where subtle pneumothoraces may be clinically significant. Hemothorax, occurring in approximately one-third of trauma patients,^33^ is also more accurately detected by CT than CXR.^20,26^ Rowan et al.^34^ demonstrated the superiority of CT in detecting pneumothorax, hemothorax, pulmonary contusions, diaphragmatic rupture, and myocardial rupture in chest trauma patients.

Despite CT’s superior diagnostic capabilities, its effect on patient management remains debated. Some studies have reported that additional findings on CT seldom alter management. For instance, one study identified 76 injuries on CT that were missed on CXR; however, only six patients had a change in management.^35^ In another study, 92 of 443 patients with blunt chest trauma had abnormal CT scans and subsequently underwent additional investigations or interventions.^20^ Conversely, other studies have reported significant changes in management in up to 70% of cases following CT scans.^35^ The impact of imaging on clinical outcomes also remains uncertain. A large retrospective analysis of the Trauma Quality Improvement Program database (comparing 17,716 chest CT with 8861 CXR patients) reported lower 24-h and in-hospital mortality among those undergoing CT; however, the survival difference was not statistically significant.^36^ A prospective German study also reported reduced mortality associated with CT.^26^ Selection bias may play a role, as hemodynamically unstable patients are more likely to undergo only CXR. In contrast, a randomized Iranian trial found no difference in survival, although the study was limited by a small sample size.^37^ These conflicting results highlight the need for larger, well-designed prospective studies to identify the optimal imaging approach for improving survival outcomes.

Although CT offers the most detailed, sensitive, and specific method for identifying thoracic injuries, its use must be weighed against increased costs and radiation exposure, especially in resource-limited settings such as Pakistan. In this study, CXR served as the primary imaging modality in most cases, while CT was performed in 13.8% of initial assessments. CT usage increased to 28.5% for the second imaging assessment, indicating its selective use for cases requiring further evaluation due to higher costs and limited availability. The increased reliance on CT reflects the need for more detailed assessment in complex or inconclusive cases after initial radiography. Given the resource constraints in Pakistan, implementing a structured triage system could help optimize imaging decisions. A severity-based protocol could prioritize initial assessment with CXR and focused assessment with sonography in trauma (FAST) ultrasound for hemodynamically unstable patients, guiding immediate surgical or procedural interventions. In stable patients, CT may be selectively utilized to evaluate suspected injuries that remain unclear after initial imaging. This approach ensures that CT scans are reserved for cases that require them the most, balancing diagnostic accuracy, optimizing cost-effectiveness, and minimizing unnecessary radiation exposure.

A decreasing trend in the number of patients with each radiological examination was also noted in our study, where nearly half of our patient cohort (n = 63; 48.5%) did not undergo a third radiological examination. Although reasons were not systematically documented as part of the study protocol, a qualitative review of available clinical notes suggested two primary contributing factors. The first, and most common, factor was appropriate clinical stabilization: initial imaging was performed during the acute phase of care, and many patients improved sufficiently to avoid further radiological follow-up before discharge. The second factor was that a subset of clinically stable patients chose to leave against medical advice. Our review indicated that this was often driven by socioeconomic challenges, including financial constraints and the logistical difficulty of returning for follow-up from remote or distant residences. This observation underscores the inherent challenges in completing longitudinal radiological follow-up in mass casualty populations, particularly in resource-limited settings and lower-middle-income countries, such as Pakistan.

This study has several strengths, including dynamic assessment of thoracic injuries across three imaging stages and detailed evaluation of injury progression over time. Conducted at a major trauma center, it offers real-world insights valuable for resource-limited regions. The relatively large sample size (130 patients) strengthens the validity and reliability of our findings. Additionally, the study focuses specifically on bomb blast injuries, a crucial yet underrepresented area in trauma research, enhancing clinical relevance and potential impact on future trauma management protocols. However, the study also has limitations. As a single-center analysis, our findings may not be fully generalizable to other populations or healthcare settings. The retrospective design introduces inherent biases, including potential gaps in medical records that may affect data accuracy. Variability in scan timing across patients may affect the assessment of injury progression, making it challenging to standardize comparisons. Finally, a key limitation of this study is the exclusive focus on radiological findings without clinical correlation. Due to the emergency protocol as per the mass casualty incident, clinical documentation and trauma scores (e.g. Injury Severity Score) were frequently not recorded. Consequently, we could not establish a direct association between the radiological findings of thoracic injuries and the patient’s clinical status. Furthermore, the lack of incorporation of the patient’s clinical course and outcome did not allow us to establish a more comprehensive picture of patient recovery and prognosis in accordance with their radiological findings.

Future studies should use prospective, multicenter designs with standardized imaging intervals, integrating validated injury severity metrics and long-term clinical outcomes to better correlate thoracic radiological progression with patient recovery. Developing validated, severity-based triage protocols that combine CXR, FAST ultrasound, and selective CT—particularly in the settings of mass casualty incidents—could optimize resource allocation in low-resource settings. Formal cost-effectiveness and radiation-risk analyses should further guide imaging strategies. Additionally, piloting AI-assisted detection algorithms on blast injury CT datasets may enhance sensitivity for subtle pneumothoraces and diaphragmatic injuries, ultimately informing evidence-based guidelines that balance diagnostic accuracy, patient safety, and healthcare efficiency.

In conclusion, bomb blast injuries pose a significant clinical challenge. In our study, foreign body injuries were the most common thoracic manifestation, emphasizing the importance of secondary trauma. Males and working-age adults are most affected due to higher exposure in public and occupational settings. Chest radiographs remain the primary imaging modality, while CT provides greater sensitivity for complex cases. However, the impact of CT on management remains debated. Future research should focus on developing standardized imaging protocols to ensure consistent assessment of thoracic injury progression and conduct prospective studies correlating injury progression and radiological findings with clinical outcomes. Refining triage protocols can optimize imaging use while balancing diagnostic accuracy with resource limitations. Evidence-based guidelines should be established to enhance trauma assessment, patient management, and overall clinical decision-making.

## Data Availability

Due to ethical restrictions, the underlying data are not publicly available but may be obtained from the corresponding author upon reasonable request and with approval from the Aga Khan University Institutional Review Board.
